# Factors associated with preterm delivery and low birth weight: a study from rural Maharashtra, India

**DOI:** 10.12688/f1000research.10659.1

**Published:** 2017-01-24

**Authors:** Anand Ahankari, Sharda Bapat, Puja Myles, Andrew Fogarty, Laila Tata

**Affiliations:** 1 Division of Epidemiology and Public Health, University of Nottingham, Nottingham, UK; 2 Halo Medical Foundation, Maharashtra, India

**Keywords:** maternal age, gravidity, birth weight, Maharashtra, India

## Abstract

**Background: **Although preterm delivery and low birth weight (LBW) have been studied in India, findings may not be generalisable to rural areas such as the Marathwada region of Maharashtra state. There is limited information available on maternal and child health indicators from this region. We aimed to present some local estimates of preterm delivery and LBW in the Osmanabad district of Marathwada and assess available maternal risk factors.

**Methods**: The study used routinely collected data on all in-hospital births in the maternity department of Halo Medical Foundation’s hospital from 1 ^st ^January 2008 to 31 ^st ^December 2014. Multivariable logistic regression analysis provided odds ratios (OR) with 95% confidence intervals (CI) for preterm delivery and LBW according to each maternal risk factor.

**Results**: We analysed 655 live births, of which 6.1% were preterm deliveries. Of the full term births (N=615), 13.8% were LBW (<2.5 kilograms at birth). The odds of preterm delivery were three times higher (OR=3.23, 95% CI 1.36 to 7.65) and the odds of LBW were double (OR=2.03, 95% CI 1.14 to 3.60) among women <22 years of age compared with older women. The odds of both preterm delivery and LBW were reduced in multigravida compared with primigravida women regardless of age. Anaemia (Hb<11g/dl), which was prevalent in 91% of women tested, was not significantly related to these birth outcomes.

**Conclusions**: The odds of preterm delivery and LBW were much higher in mothers under 22 years of age in this rural Indian population. Future studies should explore other related risk factors and the reasons for poor birth outcomes in younger mothers in this population, to inform the design of appropriate public health policies that address this issue.

## Introduction

Birth weight is an important public health indicator as it is a strong predictor of neonatal as well as lifelong health outcomes ^1^. Low birth weight (LBW) is defined as weight at birth of less than 2500 grams (<2.5 Kilograms) ^2^, which is usually associated with preterm delivery (typically less than 37 weeks of gestation) or restricted intrauterine development ^3^. Maternal factors such as nutrition, body mass index (BMI) and exposure to conditions such as malaria, tuberculosis and HIV may affect birth weight ^4^. Globally more than 20 million LBW infants (15.5% of total births) are born every year, of which about 95% are from developing countries ^2, 3^. LBW babies have a 20 times higher risk of death than babies with normal birth weight, and have a higher probability of lifetime morbidity, irrespective of ethnic differences across populations internationally ^5^.

In India it is estimated that 30% of babies are LBW, with nearly half being born full term ^3^. Whilst LBW prevalence and associated risk factors have been studied using national survey data, the generalizability of previous findings is limited due to the considerable heterogeneity between communities, particularly in rural areas. There is a sizeable population for which these data are not documented, leaving a major gap in existing literature. The Marathwada region in the state of Maharashtra has limited data on birth outcomes for its population of approximately 18 million. A recently published study using Latur District Hospital records from the Marathwada region found a LBW prevalence of 26.7% ^6^. However, no data are available for the more deprived districts of Marathwada, such as Osmanabad, which has a population of approximately 1.5 million and where the overall literacy rate is 67% (57% among females), 20% lower than the state average ^7^. Approximately 18% of the district’s population belongs to scheduled castes and tribes, recognised as being particularly deprived by the Indian government, and only 16% of the total population resides in urban areas ^7^. Healthcare access is not uniform across the region, creating further challenges in implementing routine data collection, particularly in rural and difficult to reach areas ^8^. We conducted a study to provide local estimates of preterm delivery and LBW and investigate some key maternal risk factors using hospital data from a rural Marathwada region in Maharashtra state, India.

## Methods

Halo Medical Foundation (HMF) is a non-governmental organisation (NGO) with a hospital in the Osmanabad district of Marathwada region that provides medical services to a population of nearly 100,000, spread across 60 villages ^8^. All services are provided at less than 50% of the price charged by neighbouring urban hospitals, and the hospital is attended by patients from all socioeconomic groups ^8^. We conducted a retrospective study using routinely collected data on all in-hospital births in the maternity department of HMF’s hospital from 1 ^st ^January 2008 to 31 ^st ^December 2014.

Birth weight was recorded for all live births immediately after birth under the direct supervision of an obstetrician. Low birth weight was defined as a weight of less than 2500 grams (<2.5 Kilograms) recorded immediately after birth ^3^. Determination of gestational age was based on menstrual history, clinical examination and ultrasonography investigation conducted and recorded by an obstetrician. Deliveries occurring before 37 weeks were defined as preterm ^2^. Maternal haemoglobin was measured prior to delivery by a qualified technician using the Sahli’s hemometer method (finger prick technique). This provides instant results, thus it is commonly used in the HMF hospital. Maternal anaemia was defined as haemoglobin levels of less than 11.0 g/dl ^10^.

The study used HMF hospital data retrospectively, with no communication made with doctor, patients, or any other third party for the project. The data was freely available at HMF. Thus, external approval was not deemed necessary. The HMF governance board approved this project and gave permission to use anonymised data ( Dataset 1 ^26^ ). The study is reported in accordance with the STROBE guidelines ( Supplementary Table 1) ^9^.

We restricted analyses to singleton live births, and following an initial descriptive summary of the deliveries, logistic regression analysis was conducted to investigate the association of maternal factors (age [older or younger than the mean], gravidity [primigravida or multigravida] and anaemia) with preterm delivery and, among full-term deliveries only, having a LBW baby. Results are reported as unadjusted and adjusted odds ratios (OR) with 95% confidence intervals (CI). Statistical significance was ascertained based on a p value <0.05. All analyses used the licensed statistical software package IBM SPSS (version 20).

HMF Hospital Delivery Data 2008–2014 The attached dataset includes information on maternal age, gravidity, haemoglobin levels, delivery term, and birth weight of 655 study samples. Click here for additional data file. Copyright: © 2017 Ahankari A et al. 2017 Data associated with the article are available under the terms of the Creative Commons Zero "No rights reserved" data waiver (CC0 1.0 Public domain dedication).

## Results

Throughout the study period, 685 deliveries were carried out at the hospital. After excluding missing data (n=4), twin pregnancies (n=8) and stillbirths (n=18), we analysed 655 cases of singleton live births. For these 655 cases, mean maternal age at delivery was 22 years, with 93% normal vaginal deliveries and 7% caesarean sections. The sex ratio at birth was 1.07 (males n=340, females n=315), and none of the study participants had any systemic diseases such as hypertension or diabetes, or habits which may have influenced birth weight or delivery term, such as smoking. Table 1 summarises the descriptive details of the analysed live births, 6.1% of which were preterm deliveries. All preterm deliveries were natural and none were induced by the healthcare provider. Of the full term deliveries, 13.8% were LBW babies.

**Table 1.** Characteristics of singleton live births. N=655 unless specified otherwise. SD: standard deviation.

Characteristic	Classification	Participants (N=655) (n, %)
**Maternal age**	Mean years ± SD	22.15 ± 3.17
**Gravidity**	Primigravida	337 (51.5%)
	Multigravida	318 (48.5%)
**Haemoglobin** **estimation** **performed** **on the day of** **delivery**	Yes	391 (59.7%)
	No	264 (40.3%)
	Mean haemoglobin g/dl ± SD (N=391)	9.33 ± 1.14
**Delivery term**	Full term	615 (93.9%)
	Preterm	40 (6.1%)
**Birth weight** **among** **full term** **deliveries** **(N=615)**	Low birth weight (<2.5 kg)	85 (13.8%)
	Normal birth weight (≥2.5 kg)	530 (86.2%)
	Mean birth weight kg ± SD	2.83 ± 0.44

Logistic regression analysis showed higher odds of preterm delivery in women younger than 22 years of age than in older women at the time of delivery (adjusted OR 3.23, 95% CI: 1.36 to 7.65, p=0.008) ( Table 2). Gravidity was not associated with the odds of preterm delivery. Maternal anaemia, occurring in 91% (356) of the 391 women tested, was not associated with preterm delivery. Among full term deliveries, the odds of delivering a LBW baby was twice as high in mothers who were <22 years of age at the time of delivery (adjusted OR 2.03, 95% CI: 1.14 to 3.60, p=0.02) ( Table 3). Primigravidas were two times more likely to deliver LBW babies compared with multigravidas (adjusted OR 2.87, 95% CI: 1.54 to 5.36, p=0.001). Maternal anaemia was not associated with having a LBW baby.

**Table 2.** Logistic regression analyses to assess risk factors for preterm delivery. N=655 singleton live births, unless specified otherwise. Reference category for each variable is indicated as 1.

Characteristic	Outcomes		Crude odds ratio ^ (95% CI)	Adjusted odds ratio ^ (95% CI)	p value for adjusted OR
	Preterm delivery N (%)	Full term delivery N (%)			
**Maternal age in years** **(N= 655)** **≥22 years** **<22 years**	10 (25.0) 30 (75.0)	318 (51.7) 297 (48.3)	1 3.21 (1.54 to 6.69)	1 3.23 (1.36 to 7.65) *	0.008
**Gravidity (N=655)**					
Multigravida	14 (35.0)	304 (49.4)	1	1	
Primigravida	26 (65.0)	311 (50.6)	1.82 (0.93 to 3.54)	0.95 (0.43 to 2.11) +	0.90
**Maternal anaemia status** **(N=391)**					
Not anaemic (Hb ≥ 11 g/dl)	3 (13.0)	32 (8.6)	1	1	
Anaemic (Hb < 11 g/dl)	20 (87.0)	336 (91.4)	0.64 (0.18 to 2.25)	0.61 (0.17 to 2.2) * +	0.49

^^^ : Odd ratios compare preterm with full term delivery * : Adjusted for gravidity +: Adjusted for maternal age (used as a continuous variable following linearity assessment).

**Table 3.** Logistic regression analyses to assess risk factors for low birth weight. N=615 full term singleton live births, unless specified otherwise. Reference category for each variable is indicated as 1.

Characteristic	Outcomes		Crude odds ratio ^ (95% CI)	Adjusted odds ratio ^ (95% CI)	p value
	Low birth weight N (%)	Normal birth weight N (%)			
**Maternal** **age in years** **(N=615)**					
≥22 years	24 (28.2)	294 (55.4)	1	1	
<22 years	61 (71.8)	236 (44.6)	3.17 (1.92 to 5.23)	2.03 (1.14 to 3.60) *	0.02
**Gravidity** **(N=615)**					
Multigravida	20 (23.5)	284 (53.5)	1	1	
Primigravida	65 (76.5)	246 (46.5)	3.75 (2.21 to 6.37)	2.87 (1.54 to 5.36) +	0.001
**Maternal** **anaemia** **status (N=368)**					
Not anaemic (Hb ≥ 11 g/dl)	5 (10.9)	27 (8.4)	1	1	
Anaemic (Hb < 11 g/dl)	41 (89.1)	295 (91.6)	0.75 (0.27 to 2.06)	0.75 (0.27 to 2.1) * +	0.59


^^ ^: Odd ratios compare low birth weight with normal birth weight * : Adjusted for gravidity +: Adjusted for maternal age (used as a continuous variable following linearity assessment).

## Discussion

In summary, our results show a higher likelihood of preterm delivery and having a LBW baby in women of the Marathwada region younger than 22 years of age at the time of delivery. Gravidity and anaemia were not associated with these birth outcomes.

### Strengths and limitations

This is the first study that uses data from a rural area of the Marathwada region to investigate maternal factors associated with both preterm delivery and LBW. The same obstetrician recorded all maternal health parameters and birth outcomes from in-hospital births throughout the study period. Preterm and full term deliveries were distinguished by the obstetrician through clinical examination and menstrual history and ultrasonography investigation at the time of admission. None of the study participants were diagnosed with hypertension, diabetes or other systemic conditions prior or during pregnancy, thereby limiting the influence of these confounders on our two main outcomes, LBW and preterm delivery.

The study hospital serves women across all social classes and, thus these estimates are likely to be representative of the local population in Marathwada region. However, our use of retrospective hospital records means that a detailed investigation of other maternal factors and probable confounders associated with birth outcomes is not feasible. Important factors including detailed medical history, birth spacing, maternal body mass index, education, socioeconomic status, healthcare access, knowledge and pregnancy complications which may have had important roles in our study population, were not available.

### Comparison with other studies

A community-based prospective study involving 45 villages in the Pune district of Maharashtra in the early 1990s reported that 29% of babies in the study were LBW ^11^. In the Pune study, LBW was significantly more prevalent in primiparae who were less than 20 years of age at the time of delivery than in mothers that were 21 to 25 years of age. A recent hospital based retrospective study from the southern western district of Maharashtra state investigated outcomes of teenage pregnancies (maternal age ≤19 years) ^12^. The study showed that teenage mothers were three times more likely to deliver preterm (OR 2.97, 95% CI: 2.40 to 3.70), and twice as likely to deliver a LBW baby (OR 1.80, 95% CI: 1.50 to 2.20) compared to older mothers. Findings from both studies outlined above are in agreement with our results.

However, a case-control study by Mumbare *et al* from Marathwada region reported no association between maternal age and birth weight (OR 0.53, 95% CI: 0.24 to 1.19) ^6^. The study found that a higher risk of LBW in full term delivery cases was associated with maternal weight (≤ 55 kilograms), maternal height (≤ 155 cm), weight gain during pregnancy (≤ 6 kilograms), and subsequent pregnancy spacing (<36 months). This case-control study ^6^ obtained data from two centres; the Medical College Hospital of Latur city, based in Marathwada region, and the Medical College Hospital of Nasik city, based in western Maharashtra, which has higher socioeconomic profile compared to our study population (data from July 2009 to December 2009). In this study, the mean maternal age at delivery was 23.19 years (SD: 3.37), similar to the mean age of participants in our study (22.15 years, SD: 3.17). Authors of the case-control study stated that the high prevalence of LBW (26.8%) could be because both study hospitals were tertiary care centres located in the main city of their respective districts, where high-risk pregnancy cases are referred to from surrounding villages and blocks ^6, 13^. Unlike the Mumbare *et al*, our data came from a rural hospital with comparatively low risk pregnancies (no systemic diseases or tobacco consumption were observed in our participants) ^6^.

Findings from other parts of the country also showed a higher risk of LBW and preterm delivery in younger mothers (typically defined as less than 20 years) ^14, 15^. Mean birth weight in our study was 2.83 kilograms, 16 grams higher than findings from the Karnataka study ^11^. The Karnataka study had a larger sample size (n=1138) and reported a LBW prevalence of 23%, higher than in our study. LBW prevalence of 8% to 30% reported in other Indian studies varied mainly due to study locations, sample size, hospital type (primary health centres based in villages or district hospitals based in cities), and maternal characteristics such as diet, BMI and antenatal services ^16– 21^. The recent Indian National Family Health Survey (NFHS-3) reported 34% of LBW babies at national level, with higher prevalence in rural areas compared to urban regions ^22^. Lastly, a very high prevalence of maternal anaemia (91%) among those tested was noted in our study, which is consistent with findings from other regions; however, no significant effect was seen on preterm delivery or birth weight in full term deliveries ^23^. It should be taken into account that half of the participants were tested in the week preceding delivery and the rest were tested on the day of delivery.

## Conclusion

The practice of early marriage followed by pregnancy is commonly observed in our study area. This is influenced by various factors such as parental education, financial resources, and willingness to support higher education for girls ^24^. Though the current legal age for marriage is 18 years for girls in India, child marriage remains prevalent at both state and national level ^25^. Following our observations, it may be advisable to plan the first pregnancy after 21 years of age. However this needs to be supported by necessary implementation of legislation on marriage age by the government authorities. Future studies should explore the reasons for poor birth outcomes in younger mothers in this population to inform the design of appropriate public health policies to address this issue.

## Data availability

The data referenced by this article are under copyright with the following copyright statement: Copyright: © 2017 Ahankari A et al.

Data associated with the article are available under the terms of the Creative Commons Zero "No rights reserved" data waiver (CC0 1.0 Public domain dedication). 

**Dataset 1: HMF Hospital Delivery Data 2008–2014.**

The attached dataset includes information on maternal age, gravidity, haemoglobin levels, delivery term, and birth weight of 655 study samples.

doi, 10.5256/f1000research.10659.d149854 ^26^

## Supplementary material

**Supplementary material 1: STROBE Guidelines for cross-sectional studies.**

The study is reported in accordance with the following checklist of STROBE guidelines.
