# Supplementary material for: Factors associated with preterm delivery and low birth weight: a study from rural Maharashtra, India
Source: F1000Res. 2017 Jan 24;6:72. [Version 1] doi: 10.12688/f1000research.10659.1 (PMC5428483; doi:10.12688/f1000research.10659.1)
Supplement: Peer review history

# Peer review: Referee response for version 1

**DOI:** 10.5256/f1000research.11488.r22043

**Published:** 2017-05-05

**Authors:** Jayashree Sachin Gothankar

**Affiliations:** 1 Department of Community Medicine, Bharati Vidyapeeth Deemed University Medical College, Pune, Maharashtra, India

The term 'Low risk pregnancies' is to be used carefully as it is not clear from the study that the information about absence of systemic disease is based on interpretation of tests conducted during study or history of absence of disease. If based on history then quality of data collected will be poor.

 Sahlis method for hemoglobin estimation is a less reliable method for assessment of anemia.

 To classify the birth as preterm, how were challenges to assess LMP addressed?

I have read this submission. I believe that I have an appropriate level of expertise to confirm that it is of an acceptable scientific standard.

# Response

**Published:** 2017-05-06

**Authors:** Anand Ahankari

**Affiliations:** University of Nottingham, UK

Dear Prof Gothankar, 

 Thank you very much for reviewing our paper. I have provided explanation below regarding 'Low Risk Pregnancies', which will be useful for readers. 

 Data in our study: Systematic diseases includes hypertension and diabetes mellitus (DM) were evaluated using investigations in the hospital by a gynaecologist. Serum glucose level was assessed during routine antenatal care, and blood pressure was measured at the same time. The absence of systematic disease was confirmed prior to the delivery at the hospital. 

 Data from Mumbare et al paper (ref 6): As explained in our paper, the research findings of Mumbare et al (6) used data from a district hospital (tertiary/advance healthcare facility), where high risk pregnancies were predominantly referred. However our data comes from a rural hospital where advance health services were not available thus only low risk pregnancies (with no systematic complications) were conducted at HMF's hospital. 

 I hope that readers will find this additional explanation useful. 

 Thank you once again for your valuable time. 

 Dr Anand Ahankari

# Peer review: Referee response for version 1

**DOI:** 10.5256/f1000research.11488.r19633

**Published:** 2017-02-06

**Authors:** Rahul Ramesh Bogam

**Affiliations:** 1 Department of Family and Community Medicine, King Faisal University, Kingdom of Saudi Arabia, Saudi Arabia

**Introduction**

 The present study is the retrospective analysis of hospital based data to identify some local

 estimates of preterm delivery and Low birth weight(LBW) in the Osmanabad district of Marathwada and to assess available maternal risk factors. As per author's information, this was the first study in Marathwada region of Maharashtra State to explore the information about maternal and child health indicators from this region. It is a well written manuscript with appropriate presentation of results.

 Few suggestions/ recommendations :

**Objectives / Goals**

 There is need to mention **clear/specific** objectives/ goals. In the introduction section,authors tried to mention objectives but it needs to specified. For example: 'To investigate some key maternal risk factors' can be replaced by ' To determine/ find out association of maternal risk factors with.....'. In short, objectives/goals can be re -framed.

**Methods**

 Authors have not justified the inclusion of this specific period - i.e.1st January 2008 to 31st December 2014. Authors are encouraged to provide justification for the same. Detailed inclusion and exclusion criteria need to be mentioned in METHOD section.

**Discussion**

 Strengths and limitations should be at the end of discussion section rather than at the beginning. The heading ' Comparison with other studies' may be removed from discussion section as DISCUSSION itself reflects comparison with other studies. Please make sure that all TABLES should be a part of RESULT section, not of the DISCUSSION section.

**Conclusion**

 This section can be supplemented with the heading "RECOMMENDATIONS'', or there can be separate section of recommendations as authors have given recommendations based on study findings.

**Key words**

 Authors are encouraged to provide key words for their study.

I have read this submission. I believe that I have an appropriate level of expertise to confirm that it is of an acceptable scientific standard.

# Response

**Published:** 2017-03-24

**Authors:** Anand Ahankari

**Affiliations:** University of Nottingham, UK

Dear Dr Bogam, 

 Thank you for your valuable time to review our research paper. I have provided a brief response to your comments below. 

 Regarding study objectives: In the abstract, we followed a recommended guideline of the journal, thus a separate title on the study objective was not included. The last part of the introduction is the study objective ("We conducted a study to provide local estimates of preterm delivery and LBW and investigate some key maternal risk factors using hospital data from a rural Marathwada region in Maharashtra state, India"). We are happy to re-frame this, if advised by the journal editors. 

 Regarding methods: The reason for the specific duration is mainly due to the project timeline. There is no other reason to use the give timeline. 

 Regarding discussion, conclusion and keywords: We have provided manuscript, tables and datasets seperately to the journal. The article type setting and sequence is solely managed by the journal. We submitted all files in accordance with the journal requirements. We also submitted keywords, and believe that those will appear during the final approved submission. 

 Thank you once again for your valuable time. We hope that *F1000Research* readers will find this comment section useful. 

 Dr Anand Ahankari
